# Editorial on: Bacterial pathogens in the non-clinical environment

**DOI:** 10.3389/fmicb.2015.00331

**Published:** 2015-04-21

**Authors:** Sébastien P. Faucher, Steve J. Charette

**Affiliations:** ^1^Department of Natural Resource Sciences, Faculty of Agricultural and Environmental Sciences, McGill UniversitySte-Anne-de-Bellevue, QC, Canada; ^2^Institut de Biologie Intégrative et des Systèmes, Pavillon Charles-Eugène-Marchand, Université LavalQuebec City, QC, Canada; ^3^Centre de Recherche de l'Institut Universitaire de Cardiologie et de Pneumologie de Québec (Hôpital Laval)Quebec City, QC, Canada; ^4^Département de Biochimie, de Microbiologie et de Bio-Informatique, Faculté des Sciences et de Génie, Université LavalQuebec City, QC, Canada

**Keywords:** biofilm, VBNC, protozoa, *Listeria*, *Legionella*, *Escherichia coli*, *Clostridium botulinum*, *Pseudomonas*

When thinking about bacterial pathogens, most will consider their interaction with humans. Nevertheless, many pathogens affecting humans will not be transmitted directly from one individual to another but will rather come from or transit through the environment to infect the human host. Outside their hosts, bacterial pathogens must be able to resist environmental stresses and perhaps grow in order to get to another hosts. The environment outside the host is referred therein as the non-clinical environment (NCE).

In this research topic, a collection of articles is presented that covers some of the strategies and factors that influence the survival and growth of bacterial pathogens in the NCE, and therefore affects transmission to humans, and outbreaks. Such knowledge could be important to limit the transmission during an outbreak. For example, a Legionnaires' disease outbreak in Quebec City (Canada) in 2012 prompted Trudel et al. (2014) to review the effort to find the source. They conclude that better collaboration between government agencies, academia, and the industry could prove beneficial in the fight against bacterial infections.

Importantly, bacterial pathogens will require adapting their genome to persist and grow in the NCE. This may affect the interaction with human hosts, as stated by Dr. Martinez: “Evolution of human pathogens is not exclusively driven by the infection of human” (Martínez, 2014). To support his opinion, the author gives the example of *Yersinia pestis* who has lost genes to kill its insect host, which allowed for a better transmission between animal hosts, including human by using the insect as a vector. Martinez also discusses the concept of short-sighted evolution during infection.

Evolution of transmission properties is an important aspect of pathogens coming from the NCE. To illustrate that, Leong et al. (2014) studied the presence of *Listeria monocytogenes* in several processing plants in Ireland and on the food produced by those plants. They showed that some pulsotypes were commonly found in several different facilities. Some strains are likely better equipped to persist in the facilities and consequently contaminate food more frequently (Leong et al., 2014).

In the NCE, the pathogens will also interact with other microorganisms, invertebrates and plants, which may all shape how the pathogens behave as well as its ability to infect human hosts. Growth of bacterial pathogens in the NCE may allow them to reach concentrations that ensure their transmission to new hosts. The complex relationship between the NCE and outbreak occurrence is very well illustrated by *Clostridium botulinum* (Espelund and Klaveness, 2014). The authors suggest that accumulation of *C. botulinum* spores in carcasses, algal mats and biomass, and further bioaccumulation of the toxin is central in causing diseases.

In the NCE, bacterial pathogens will encounter protozoa. These are ubiquitous unicellular eukaryotes and many feed on bacteria. Therefore, there is an evolutionary pressure on bacteria to resist grazing by protozoa. Some, such as *Legionella pneumophila*, have evolved a strategy to highjack them and grow intracellularly. In their review, Robertson et al. (2014) point out that *L. pneumophila* has a far more complex developmental cycle than normally thought which includes the production of mature infectious forms by amoebae thought to increase the potential of transmission of *L. pneumophila* from water systems to the human host (Robertson et al., 2014).

Some species of protozoa are known to produce and expel vesicles while grazing on bacteria. These vesicles, sometime referred to as pellets, may contain live bacteria (Denoncourt et al., 2014). Packaging of bacteria by protozoa increases their resistance to biocides and other environmental stresses. In addition, pellets may increase infectivity of bacterial pathogens. The authors, based on the literature, thus propose the hypothesis that packaging of bacterial pathogens by protozoa is important for their persistence and for their transmission to human and animal hosts (Denoncourt et al., 2014).

Bacteria exposed to stresses in the NCE have evolved strategies to deal with them. Therefore, bacterial pathogens have learned tricks in the NCEs that prove to be efficient to promote infectious diseases. Biofilm is important for the persistence of bacterial cells in the NCE, since bacterial cells inside biofilm are more resistant to biocides and stressful conditions than planktonic cells. Vogeleer et al. (2014) discuss the role of biofilm for the persistence of Shiga-toxin producing *E. coli* (STEC) in the NCEs, including soil, water systems, meat processing plants, and on fresh produce. They argue that STEC biofilm are likely an important source of contamination of finished products and a concern for public health (Vogeleer et al., 2014).

The viable-but-not-culturable (VBNC) state is characterized by live and metabolically active cells, but unable to grow on standard laboratory medium (Li et al., 2014). This can complicate the detection of bacterial pathogens in water, food, and from infected tissues. This state can be triggered by a variety of stressful conditions frequently encountered in NCE. VBNC cells are notoriously more resistant than culturable cells to physical and chemical stresses. In addition, resuscitation of VBNC cells can occur in conditions permissive for their growth or when exposed to their hosts. Many genetic factors are involved in the induction of the VBNC state and resuscitation from it, but we are still far away from being able to adequately detect cells in this state and develop ways to avoid them (Li et al., 2014).

Persisters are non-growing phenotypic variant of a population that are tolerant to antibiotic (Amato et al., 2014). They are genetically identical to the rest of the population; only their physiological state is different. Persistence has probably evolved in response to antibiotic producing microbes in the NCE. This state has clinical importance for the treatment of infectious diseases: following an antibiotic treatment, a small proportion of the population will survive and when the antibiotic fades away, the survivors resume growth. Efforts are needed to find drugs to block persistence during antibiotic treatment (Amato et al., 2014).

The articles published in this research topic clearly highlight that the behavior of bacterial pathogens and their interaction with other organisms in the NCE influence their transmission and their performance during infection. A complete understanding of virulence and epidemiology and the development of effective countermeasures against bacterial pathogens would be ultimately successful only if their whole life cycle, including their life in the NCE, is taken into account.

## Conflict of interest statement

The authors declare that the research was conducted in the absence of any commercial or financial relationships that could be construed as a potential conflict of interest.
